# Bearing Fault Diagnosis via FMD with Parameters Optimized by an Improved Crested Porcupine Optimizer

**DOI:** 10.3390/s25237339

**Published:** 2025-12-02

**Authors:** Ping Pan, Hao Liu, Bing Lei, Xiaohong Tang

**Affiliations:** 1 School of Mechatronics and Vehicle Engineering, East China Jiaotong University, Nanchang 330013, China; ppping0210@126.com (P.P.); 13407060551@163.com (H.L.); 2 School of Intelligent Technology, Jiangxi Open University, Nanchang 330046, China; leibing1992@126.com

**Keywords:** rolling bearing, feature mode decomposition, improved crested porcupine optimizer, fault diagnosis, feature extraction

## Abstract

Feature Mode Decomposition (FMD) can effectively extract bearing fault features even in the case of strong interference noise by means of adaptive finite impulse response filter banks along with correlated kurtosis. Nevertheless, the filter length *L* and the number of decomposition modes *K* need to be predefined carefully in a manual way. Otherwise, mismatched parameters could lead to redundant components or even missed detection of fault information. To mitigate the reliance on manual parameter setting, recent studies have introduced optimization algorithms such as the Whale Optimization Algorithm and the Crested Porcupine Optimizer to find the optimal parameters for FMD. However, such methods usually suffer from the dilemma of easily premature convergence in global search and long-time consumption in local fine adjustment, rendering them with difficulty in meeting the requirements of real-time and accurate diagnosis. Therefore, this paper proposes an improved Crested Porcupine Optimizer (ICPO), which can dynamically balance global and local exploitation. Furthermore, a bearing fault diagnosis method named ICPO-FMD is constructed, wherein the optimal parameter combination of *K* and *L* obtained using ICPO is provided to FMD in order to decompose bearing signals into a family of intrinsic mode functions (IMFs), and then fault sensitive components are extracted according to the proposed IMF screening principle. Finally, a reconstructed signal is obtained, followed by an envelope demodulation analysis. Experiments on simulation, laboratory and engineering signals demonstrate that the proposed method can accurately extract the fault characteristic frequency and its harmonics.

## 1. Introduction

In the context of rolling bearing fault diagnosis, signal processing techniques have evolved from traditional time-domain and frequency-domain methods to advanced time-frequency analysis approaches [1]. Among various signal decomposition techniques, adaptive methods such as Empirical Mode Decomposition (EMD) and its variants (EEMD, CEEMDAN) [2,3,4] can decompose signals without preset basis functions, yet they suffer from mode aliasing issues that limit their effectiveness in complex fault scenarios.

Recent studies have explored diverse approaches to enhance fault diagnosis performance. Qian et al. [5] proposed a cyclostationarity-based multi-band demodulation method for bearing fault detection, and Wen et al. [6] developed a robust anomaly detection framework capable of handling contaminated data. In addressing challenges under variable working conditions, Sprito et al. [7] enhanced the SDP-CNN framework by integrating Multi-Order Tracking Filtering for gear fault detection, demonstrating that Symmetrized Dot Pattern (SDP) transformation combined with Order-Tracking techniques and Convolutional Neural Networks can effectively handle vibration signals under stationary and time-varying operating conditions, achieving superior classification accuracy and robustness compared to classical approaches. In parallel, the emergence of domain adaptation techniques has addressed the challenge of transferring diagnostic knowledge across different operating conditions. Notably, Wang et al. [8] proposed a domain-reinforced feature adaptation method with correlation alignment for compound fault diagnosis of rolling bearings, which effectively mitigates distribution discrepancies between source and target domains through adversarial training mechanisms. This approach demonstrated significant improvements in cross-domain fault recognition accuracy, particularly under variable speed and load conditions where traditional methods struggle. Furthermore, recent advances in intelligent optimization have introduced meta-learning frameworks for autonomous fault identification. Wang et al. [9] developed a reinforcement adversarial open-set algorithm for autonomous recognition of compound faults in mechanical equipment, which combines reinforcement learning with adversarial training to handle unknown fault categories without requiring exhaustive labeled data. Their framework achieved robust performance in scenarios with incomplete fault knowledge, addressing a critical limitation of supervised learning approaches that assume closed-set fault taxonomies.

Despite these advances, inherent limitations persist: multi-band demodulation methods necessitate manual tuning of multiple parameters (e.g., decomposition levels, thresholds); domain adaptation techniques often demand substantial quantities of target-domain data to achieve satisfactory transfer performance; and reinforcement learning frameworks face challenges in sample efficiency and convergence stability under high-dimensional feature spaces. Furthermore, Variational Mode Decomposition (VMD) was proposed by Dragomiretskiy et al. [10]. While VMD-based optimization methods [11,12] have attempted to automate parameter selection through intelligent algorithms, they continue to struggle with intrinsic parameter sensitivity and lack feature-guided decomposition mechanisms that can autonomously target fault-sensitive characteristics. More critically, existing methods seldom integrate adaptive signal decomposition with intelligent optimization in a unified framework that simultaneously addresses parameter automation, feature sensitivity, and computational efficiency—a gap that motivates the present study.

Against this background, Miao et al. [13] proposed Feature Mode Decomposition (FMD), which takes a different approach: using the correlated kurtosis (CK) as the optimization objective, FMD can adaptively lock onto the impulse components without prior frequency band hints, achieving more thorough IMF separation. Experiments show that its robustness in strong noise scenarios is significantly better than that of VMD. However, the number of modes and filter length of FMD still rely on manual setting, and parameter mismatch can also lead to component redundancy or information omission. Therefore, establishing a data-driven FMD parameter optimization strategy has become a key step to broaden its engineering applications.

In the past two years, scholars have employed various intelligent algorithms to optimize the parameters of FMD. Wang et al. [14] utilized the Whale Optimization Algorithm (WOA) to simultaneously search for the optimal modal number *K* and filter length *L* of FMD. However, the spiral update mechanism of WOA is prone to getting stuck in local extremums in a multidimensional parameter space, resulting in a rapid reduction of the “hunt” range in the later stages of convergence, leading to premature convergence. Yan et al. [15] used Particle Swarm Optimization (PSO) to optimize the two parameters of FMD. During the iterative process, although it has good global search capabilities, the population diversity will be lost relatively quickly, which may lead to premature convergence of the algorithm. In summary, the existing optimization algorithms are either “early-maturing” or “slow-maturing”. Fortunately, the Crested Porcupine Optimizer (CPO) proposed [16] at the end of 2024 precisely fills this gap. The “sight-sound-odor” triple perception mechanism of the Crested Porcupine can be abstracted as a three-stage search process of “global scanning + local raid + random perturbation”. This not only avoids local traps but reduces the number of iterations to less than half of that of traditional algorithms. Nevertheless, the naive CPO is not perfect either in the sense that if the warning radius is reduced too rapidly, in the later stages, it may still “over-search” and miss the better solution; if the population initialization is concentrated in the same area, it will slow down the convergence. As such, this paper has made three improvements to the naive CPO, as described in the main text. These three improvements enable CPO to “be fast first and then slow”, balance the overall and local aspects, and also ensure the uniform distribution of the initial population and enhance diversity. The improved algorithm is named ICPO (improved CPO), which is employed to jointly optimize *K* and *L* of the FMD. Eventually, the ICPO-FMD based bearing fault diagnosis framework is obtained. Experiments show that compared with other methods such as CPO-FMD and ICPO-VMD, the ICPO-FMD-based method not only maintains the same diagnostic accuracy but also significantly improves its stability under strong noise conditions. The main contributions of this paper are as follows:

(1) An improved Crested Porcupine Optimizer is developed with fast-to-slow search and global–local dynamic balance, which effectively enhances convergence efficiency and population diversity.
(2) A novel ICPO-FMD framework is constructed, where ICPO adaptively optimizes the filter length *L* and the number of modes *K* in FMD, and a fault-sensitive IMF screening principle is introduced to ensure accurate feature extraction.
(3) Experimental validation on simulated, laboratory, and engineering datasets demonstrates that ICPO-FMD achieves robust and stable fault diagnosis.

The remainder of this paper is organized as follows: Section 2 presents the theoretical foundations of the proposed method. Section 3 introduces the ICPO-based FMD parameter optimization framework and elaborates on the IMF selection strategy. Section 4 validates the effectiveness of the proposed method using simulated signals, laboratory signals, and engineering signals. Finally, Section 5 concludes the paper and discusses potential future research directions.

## 2. Methods

### 2.1. Feature Mode Decomposition

FMD can effectively highlight the bearing fault impacts, which are often masked by strong background noise. The main steps of FMD are as follows:

Step 1: Load the original vibration signal x and input the parameters of FMD, including the number of decomposed modes *K* and the filter length *L*.

Step 2: Initialize the FIR filter group with Hanning windows using *G* filters $f_{g}^{i}$ and set the iteration counter *i* = 1. It should be noted that the FMD method requires *G* ≥ *K*, but an excessively large *G* value will affect its computational efficiency.

Step 3: Obtain the filtered signal using $u_{g}^{i}$ = x ∗ $f_{g}^{i}$, where g = 1, 2, …, *G*, and ∗ represents convolution operation, $u_{g}^{i}$ is the decomposed mode component.

Step 4: Update the filter coefficients using the original signal x, the decomposed mode components $u_{g}^{i}$, and the estimated fault period $T_{g}^{i}$. The estimated fault period $T_{g}^{i}$ is determined by the local maximum $R_{g}^{i}$ after the autocorrelation function of $u_{g}^{i}$ go across the zero-crossing point. The iteration counter *i* is incremented by *i* = *i* + 1.

Step 5: Determine whether the current iteration count *i* reaches the maximum iteration count. If not, return to step 3; otherwise, continue to step 6.

Step 6: Construct a correlation matrix $CC_{\left(G*G\right)}$, calculate the correlation coefficient between each pair of mode components, select the two mode components with the maximum correlation coefficient, followed by calculating their correlated kurtosis using the previously estimated fault period $T_{g}^{i}$. Then, select the mode component with the larger kurtosis as the decomposed mode component, and set the filter number *G* = *G* − 1.

Step 7: If the current filter number *G* is equal to the set number of decomposed modes K, the iteration is stopped and K mode components are obtained as the final result of FMD; otherwise, return to step 3.

The two input parameters of FMD (i.e., the number of decomposed modes *K* and the filter length *L*) have a significant impact on its performance. Improper decomposed modes *K* may lead to over-decomposition or mode aliasing, while inappropriate filter length *L* may affect the filtering performance of the filter [17]. Therefore, it is urgent to optimize the two parameters of FMD to improve its fault feature extraction ability, especially in the presence of strong background noise, and the key to parameter optimization lies in an appropriate evaluation index.

### 2.2. Improved Crested Porcupine Optimizer (ICPO)

Although the CPO demonstrates promising global search capability, it still suffers from several limitations that hinder its performance in practical applications. First, the initialization process does not fully exploit the four defense mechanisms of crested porcupines, leading to an initial population that lacks a natural link with the subsequent search strategy. In addition, the use of fixed initialization parameters restricts the adaptability of CPO to different optimization problems, resulting in insufficient population diversity and uneven coverage of the solution space. Furthermore, the absence of effective mechanisms for group cooperation and information exchange reduces the efficiency of collective learning. Another limitation lies in the fixed control parameters, which cannot adaptively adjust the balance between exploration and exploitation across different optimization stages, thereby accelerating the decline of population diversity and increasing the risk of premature convergence. Finally, while CPO achieves satisfactory results on low-dimensional benchmark functions, its convergence efficiency and robustness degrade significantly in high-dimensional and complex optimization scenarios, making it difficult to escape from local optima and approach the global optimum. To overcome these drawbacks, this paper introduces several strategies to enhance the naive CPO, thereby improving both convergence efficiency and solution quality.

#### 2.2.1. Nonlinear Forward Adaptive Initialization

To enhance the quality and diversity of population initialization, a Nonlinear Forward Adaptive Initialization (NFAI) framework is proposed. This framework introduces a multi-stage collaborative mechanism that not only improves the quality of the initial population but also ensures its uniform distribution across the search space. The overall process can be expressed as:

(1) $$X_{init}=\Phi \left(X_{base},X_{enhanced},F,\theta \right)$$

where $X_{base}\in R^{N_{b}\times D}$ represents the base population, $X_{enhanced}\in R^{N_{e}\times D}$ represents the enhanced population, F is the objective function, and θ denotes the set of control parameters. The NFAI framework consists of four major stages: base population generation, guiding point identification, enhanced population construction, and iterative pre-optimization, followed by final population selection.

(1) Base population generation

The initial candidate solutions are generated as:

(2) $$X_{base}^{i}=lb+r_{i}⊙\left(ub-lb\right),\;i=1,2,\ldots ,N_{b}$$

where $r_{i}\in [0,1{]}^{D}$ is a uniformly distributed random vector, ⊙ represents the Hadamard product, and *lb*, *ub* are the lower and upper bounds of the search space.

(2) Guiding point identification

A set of guiding points is identified by fitness evaluation:

(3) $$G=X_{i}|F\left(X_{i}\right)\leq F\left(X_{j}\right),\forall_{j}\in 1,2,\ldots ,N_{g}$$

(4) $$N_{g}=max\left(3,\left\lfloor 0.1\cdot N_{b}\right\rfloor \right)$$

where $G\subset X_{base}$ denotes the set of guiding points, $N_{g}$ represents the number of selected guiding points, and it represents the top 10% of the individuals with the best fitness in the population. These points serve as anchors to guide the subsequent enhancement process.

(3) Enhanced population construction

Two complementary strategies are employed to simulate the defensive mechanisms of crested porcupines.

- *Local reverse learning strategy*: This mechanism exploits the symmetry of the search space to explore potential high-quality regions [18]:
  (5) $$X_{i}^{new}=M+\beta \cdot \left(M-X_{i}\right)$$
  (6) $$M=\frac{ub+lb}{2}$$
  (7) β=0.3+0.5⋅rand()
  where *M* is the search space center and *β* is the reflection coefficient.
- *Elite fusion strategy*: High-quality individuals are adaptively fused to accelerate convergence towards promising regions [19]:
  (8) $$X_{i}^{new}=\sum_{j=1}^{N_{e}}w_{j}X_{g_{j}}+\gamma \cdot N\left(0,\sigma^{2}\right)⊙\left(ub-lb\right)$$
  where $w_{j}$ is the adaptive weight coefficient, satisfying $\sum_{j=1}^{N_{e}}w_{j}=1$, γ is the perturbation intensity parameter, and $X_{g_{j}}\in G$ is the elite individual.

The weight coefficient can be calculated by:

(9) $$w_{j}=\frac{e^{-\eta \cdot F\left(X_{g_{j}}\right)}}{\sum_{k=1}^{N_{e}}e^{-\eta \cdot F\left(X_{g_{k}}\right)}}$$

where η is the regularization coefficient, which gives greater weight to the elite solutions with better fitness.

(4) Iterative pre-optimization

To further refine the population, an iterative pre-optimization process [20] is introduced. In iteration k−th, the adaptive scaling factor $\lambda_{k}$ is dynamically updated:

(10) $$\lambda_{k}=\lambda_{0}\cdot \left(1-\lambda_{0}\cdot \frac{k}{T_{max}}\right)$$

where $\lambda_{0}=0.5$ represents the initial factor value. Candidate solutions are then transformed via a hybrid probabilistic distribution strategy:

(11) $$X_{i}^{t+1}=\left\{\begin{array}{l} \min(\max(X_{i}+S(t)f_{\alpha tan}(R(t)r_{1})⊙(X_{gb}-X_{i}),l_{b}),u_{b})\\ X_{i} \end{array}\right.$$

where the search vector $r_{1}$ adopts a three-state mixed distribution generation mechanism to ensure the coverage of the population:

(12) $$r_{1}^{d}=\left\{\begin{array}{l} N(0,1.0^{2}),\;if\;\xi_{1}\;<0.5\\ 0.2\cdot sign(N(0,1)),\;if\;\xi_{1}\;\geq 0.5\;and\;\xi_{2}\;<0.3\\ sign(N(0,1))\cdot (0.4+0.4\xi_{3}),\;if\;\xi_{1}\;\geq 0.5\;and\;\xi_{2}\;\geq 0.3 \end{array}\right.$$

The nonlinear transformation is achieved through dynamic search of radius *R*(*t*) and the arctangent function $f_{atan}$. Mathematically, it can be expressed as:

(13) $$R\left(t\right)=4.0-0.5t$$

(14) $$f_{\alpha tan}\left(r_{r}\right)=\frac{2}{\pi }\arctan(\frac{\pi R\left(t\right)r_{1}}{2})$$

where *η*, *ξ*_1_ *ξ*_2_ *ξ*_3_ ∈ U (0, 1) are independent uniform random variables, *S*(*t*) is the dynamic scaling factor. This design enables adaptive exploration of multimodal landscapes.

(5) Final population selection

Finally, a two-level strategy [21] balances quality and diversity: 70% of the population is selected from elite individuals, while the remaining 30% is determined by a max–min distance criterion to ensure spatial uniformity:

(15) $$X_{final}=X_{e}\cup X_{d}$$

where $X_{e}$ represents the elite subset selected based on quality, and $X_{d}$ is the diversity subset selected based on the maximum-minimum distance. For each individual in the diversity subset, select the candidate solutions that meet the following conditions:

(16) $$X_{d}^{j}=\arg\underset{X_{i}\notin X_{e},X_{k}\in X_{e}UX_{d}^{1:j-1}}{max}\min\;\;\left|\left|X_{i}-X_{k}\right|\right|$$

This formulation guarantees that each individual in the diversity subset maintains the maximum possible minimum distance from the selected individuals, thereby enhancing both the uniformity and diversity of the population distribution.

#### 2.2.2. Uniformly Introduce the Optimal Individuals

In the traditional CPO, the second defense mechanism generates new solutions by taking the mean of two randomly selected individuals, which restricts the information exchange to a low-dimensional subspace and may reduce search diversity. To address this limitation, and inspired by the multi-point mutation strategies proposed by Wu et al. [22] and Jia et al. [23], a multi-level information fusion strategy is introduced in this study. Specifically, a three-point mutation scheme is adopted by incorporating the current best solution as an additional reference point, thereby enhancing both exploration and exploitation. The updated solution can be expressed as:

(17) $$y=\frac{X_{Gb}+X_{i}+X_{r}}{3}$$

(18) $$X_{i}^{new}=U_{1}⊙X_{i}+\left(1-U_{1}\right)⊙\left(y+\alpha \cdot \left(X_{r_{1}}-X_{r_{2}}\right)\right)$$

where $U_{1}\in 0,1^{D}$ is a binary mask vector, and $X_{r}$, $X_{r_{1}}$, $X_{r_{2}}$ are three randomly selected individuals from the population.

#### 2.2.3. Porcupine Information Exchange Strategy

To overcome the premature convergence and insufficient adaptability of the original CPO, this study develops a nonlinear adaptive search strategy that integrates dynamic parameter control and multi-modal sampling. The mechanism is composed of four modules: nonlinear attenuation, multi-modal hybrid distribution sampling, dynamic search adjustment, and nonlinear mapping for position updating.

(1) Nonlinear attenuation mechanism

A nonlinear attenuation factor *k*(*t*) and an iterative phase function *φ*(*t*) are introduced to regulate the search capability along the iteration process:

(19) $$k\left(t\right)={\left(1-\frac{t}{T_{max}}\right)}^{1.2}$$

(20) $$\phi \left(t\right)=\frac{t}{T_{max}}$$

where *t* denotes the current iteration and $T_{max}$ is the maximum iteration. With increasing k(t) gradually decreases, guiding the algorithm to emphasize global exploration in the early stage and local exploitation in the later stage.

(2) Multi-modal hybrid distribution sampling

To balance exploration and exploitation in complex search spaces, a multi-modal sampling mechanism is designed by combining Gaussian distributions with different bandwidths and piecewise uniform distributions. In a *D*-dimensional space, the perturbation vector of each dimension integrates the fine local search ability of a narrow-band Gaussian distribution (standard deviation δ=0.4), the broader exploration of a wide-band Gaussian (δ=1.2), and the coverage expansion of multi-interval uniform sampling. This hybrid sampling significantly enhances diversity and robustness, preventing premature convergence to local optima.

(3) Dynamic search mechanism

To further enhance the adaptability of the algorithm, a dynamic search radius *R*(*t*) and scale factor *S*(*t*) are further introduced:

(21) $$R\left(t\right)=4+2\sin\left(\phi \left(t\right)\pi \right)$$

(22) $$S\left(t\right)=2\left(1-0.5\phi \left(t\right)\right)$$

where *R*(*t*) oscillates periodically within [4,6], enabling adaptive adjustment of the search range, while *S*(*t*) decreases linearly from 2 to 1 to strengthen fine-grained local exploitation in the later phase.

(4) Nonlinear mapping and position update

Finally, to ensure rationality of step-size adjustment, a nonlinear mapping based on the arctangent function is adopted [24]:

(23) $$f_{\alpha tan}\left(x\right)=\frac{2}{\pi }\arctan(\frac{\pi x}{2})$$

The individual position is updated as:

(24) $$X_{i}^{t+1}=X_{i}^{t}+S\left(t\right)r_{1}\cdot f_{\alpha tan}(R\left(t\right)r_{1})\cdot (X_{gb}-X_{i}^{t})$$

where $X_{i}^{t}$ represents the position of the i−th individual at time *t*, and $X_{gb}$ is the global best solution. This nonlinear fusion strategy enables the algorithm to maintain strong global search capacity while ensuring stable convergence, making it particularly effective for high-dimensional problems with multiple local optima.

### 2.3. Performance Evaluation of the ICPO Algorithm

To rigorously assess the effectiveness of the proposed ICPO algorithm, we conducted a series of tests using 29 standard benchmark functions from the CEC2017 test suite. The performance of ICPO was compared against eight other state-of-the-art algorithms: CPO, TOC, SFOA, SABO, ZOA, DBO, SSA, and GJO. The benchmark test functions are summarized in Appendix A. The test functions are categorized as follows:

- F1, F3: Unimodal functions, designed to evaluate the convergence speed and accuracy.
- F4 to F10: Multimodal functions, assessing the global search ability of the algorithms.
- F11 to F20: Mixed functions, used to test the adaptability of the algorithms in complex environments.
- F21 to F30: Combined functions, focusing on performance evaluation in highly non-convex and multimodal optimization problems.

All algorithms were tested under identical experimental conditions:

- Operating System: Windows 10
- Programming Environment: MATLAB R2024a
- Hardware Configuration: Intel i5-8250U processor with 20GB of memory

For the sake of fairness, the parameters for all algorithms were standardized:

- Population size: 100
- Dimensionality: 100
- Maximum number of iterations: 1000
- Repetitions: Each algorithm was run 30 independent trials
- Evaluation Metrics: Optimal value, mean value, standard deviation, P-value, and algorithm ranking

**Figure 1:**
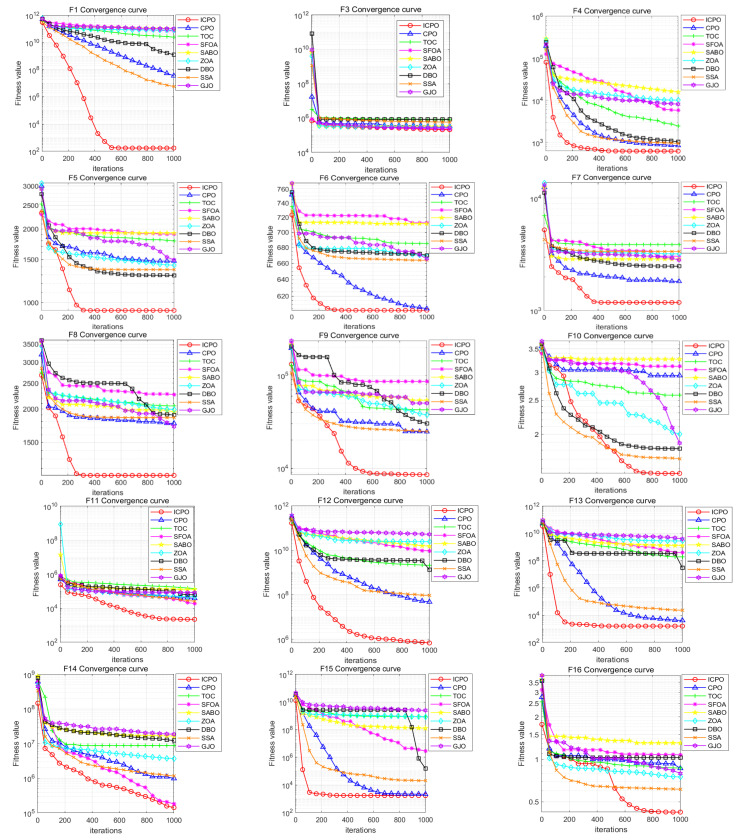

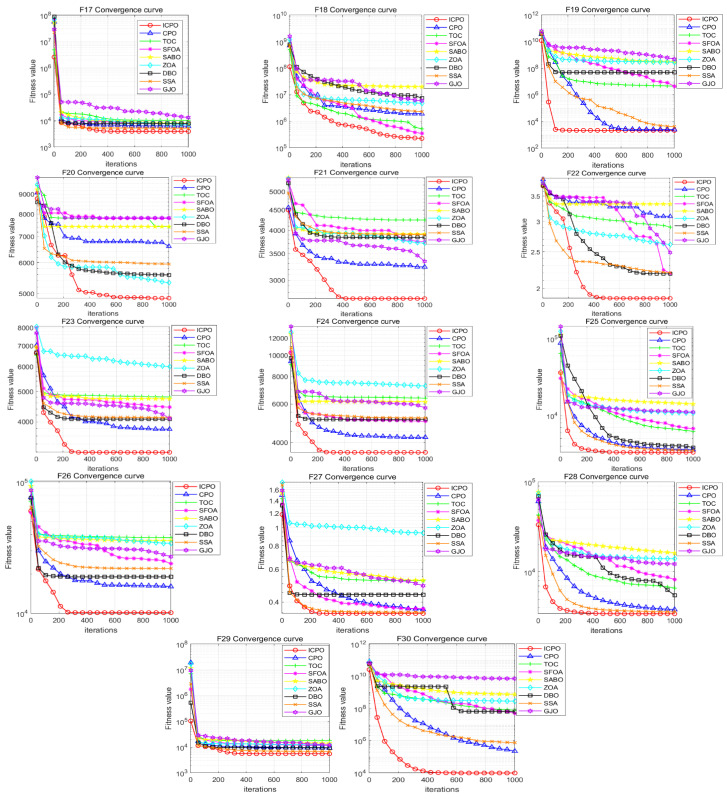
The convergence curves for different test functions.

The convergence curves displayed in Figure 1 demonstrate that ICPO outperforms the CPO algorithm in 26 out of a total of 29 test functions in terms of convergence accuracy. In particular, ICPO shows an almost an order-of-magnitude improvement over CPO in several functions, including F1 and F12. The superior local optimum escape capability of ICPO is particularly evident in the convergence curves. For example, in functions such as F5, F8, F9, and F22, ICPO manages to escape local optima during the later stages of the iterations and continues global exploration, whereas other algorithms converge prematurely, failing to find better solutions. This highlights that ICPO significantly enhances optimization capability by leveraging the synergistic effect of multiple strategies.

Appendix B shows the statistical optimization results of each algorithm on the 100-dimensional test functions. The results reveal that ICPO outperformed the CPO algorithm in terms of the mean results for 26 out of 29 test functions, achieving a performance improvement rate of 89.7%. In functions such as F6, F15, and F19, although the mean value of ICPO was slightly inferior to CPO, its optimal value surpassed that of CPO, implying that ICPO excels in extreme value optimization. For functions like F3, F14, and F18, the SFOA algorithm exhibited strong performance, ranking first among the algorithms, with ICPO following closely behind. In functions such as F10, F20, and F22, despite ICPO’s slightly lower mean values compared to ZOA, DBO, and SSA, its optimization ability outperformed these algorithms.

The Wilcoxon rank-sum test further confirms that ICPO achieves statistically significant improvements over CPO (*p* < 0.05) in the 29 tested functions. This statistical validation emphasizes that the multi-strategy improvements incorporated into ICPO effectively mitigate the issue of premature convergence, leading to a substantial enhancement in overall optimization performance.

The comprehensive experimental results demonstrate that ICPO consistently outperforms the standard CPO and other benchmark algorithms, establishing it as a robust and effective approach for function optimization tasks and providing a novel solution for FMD parameter optimization.

## 3. Parameter-Optimized FMD Based on the ICPO

### 3.1. Method Framework

In this section, we propose the ICPO-FMD algorithm, which utilizes the improved CPO to optimize the key parameters of the feature modal decomposition method, namely the filter length (*L*) and the number of decomposition modes (*K*). The parameters set in the improved Crested Porcupine Optimizer are as follows: Pop_size = 15; Tmax = 20; lb = [3,10]; ub = [50, cutnum]; Where “cutnum” represents the set parameter.

**Figure 2:**
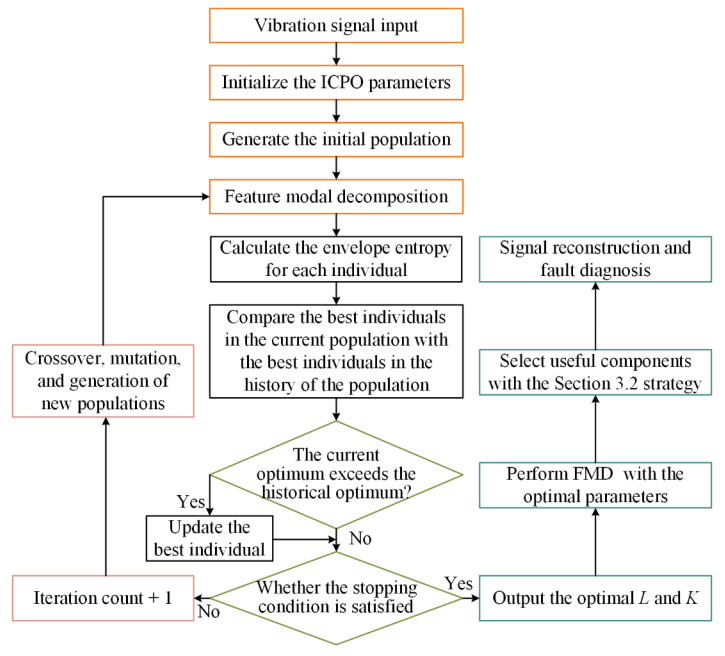
ICPO-FMD diagnostic flowchart.

The flowchart of ICPO-FMD is shown in Figure 2, and the specific steps are as follows:

First, ICPO searches the parameter space of *L* and *K*, exploring potential values to achieve the minimum envelope entropy. Through its multi-strategy optimization mechanism, ICPO balances global exploration and local exploitation, ensuring efficient convergence to the optimal parameter set for each vibration signal.

Once the optimal values of *L* and *K* are identified, these parameters are used as input for the FMD process. The vibration signal is decomposed into a set of IMFs based on the optimized parameters, which reflect the intrinsic oscillatory modes of the signal at hand. The decomposition provides more meaningful frequency components, enhancing the quality of fault feature extraction.

The overall process consists of the following steps:

(1) Optimization: ICPO optimizes *L* and *K* by minimizing envelope entropy.
(2) Decomposition: The vibration signal is decomposed into IMFs using the optimized parameters.
(3) Useful component extraction: The useful components related to the fault are selected for signal reconstruction based on the component selection strategy outlined in Section 3.2.
(4) Fault diagnosis: The reconstructed signal is subjected to envelope analysis to extract fault characteristic frequencies and identify the fault type.

### 3.2. Informative IMF Selection Strategy

The FMD process decomposes vibration signals into a set of IMFs, which differ in fault-related information, noise content, and redundancy. To ensure accurate bearing fault diagnosis, it is essential to identify and retain the IMFs that carry substantive diagnostic features. In this study, an IMF selection strategy is developed by integrating time-domain and frequency-domain indicators into a comprehensive scoring model, enabling effective evaluation and selection of informative IMFs.

#### 3.2.1. Time-Domain Indicators

Time-domain measures characterize the impulsive morphology of each IMF via its Hilbert envelope e(t). Let N denote the envelope length, the mean $u_{e}$ and root-mean-square (RMS) can be calculated by:

(25) $$\left\{\begin{array}{l} u_{e}=\frac{1}{T}\sum_{t=1}^{T}e\left(t\right)\\ RMS_{e}=\sqrt{\frac{1}{T}\sum_{t=1}^{T}e^{2}\left(t\right)} \end{array}\right.$$

Two metrics are used:

Shape Factor (SF):

(26) $$SF=\frac{RMS_{e}}{u_{e}},\left(u_{e}>0\right)$$

Crest factor (CF):

(27) $$CF=\frac{\max(e(t))}{RMS_{e}}$$

Higher SF/CF values indicate stronger impulsive content and greater likelihood that the IMF contains fault impacts

#### 3.2.2. Frequency-Domain Metrics

Frequency-domain measures are computed from the normalized envelope spectrum *E*(*f*), sampled at frequency *f_k_*, *k* = 1, …, *N_f__._*

Envelope-spectrum kurtosis (EPK)—used as an impulsiveness indicator of the envelope spectrum:

(28) $$EPK=\frac{1}{N_{f}}\sum_{k=1}^{N}\left[{\left(\frac{E_{k}-\mu_{E}}{\sigma_{E}}\right)}^{4}\right]$$

where $\mu_{E}$ and $\sigma_{E}$ are the mean and standard deviation of {*E_k_*}.

Energy concentration (EC)—measures spectral localization of energy around the centroid [25].

(29) $$\left\{\begin{array}{l} f_{c}=\frac{\sum_{k=1}^{N}f_{k}\cdot E_{k}^{2}}{\sum_{k=1}^{N}E_{k}^{2}}\\ \Delta f=\sqrt{\frac{\sum_{k=1}^{N}(f_{k}-f_{c}{)}^{2}\cdot E_{k}^{2}}{\sum_{k=1}^{N}E_{k}^{2}}}\\ EC=\frac{1}{\Delta f+\varepsilon } \end{array}\right.$$

where *ε* is a small constant to avoid division by zero. Larger *EC* means energy is more tightly concentrated.

Harmonic clarity (HC)—quantifies the presence of expected characteristic harmonics. Given a nominal fault frequency *f*_0_ and a set of harmonics $\left\{hf_{0}:h=1,\cdots ,H\right\}$, for each harmonic search for the local maximum *A_h_* in $\left|f-hf_{0}\right|<\Delta f$ (Δf = 3 Hz). Let $N_{med}$ = median{*E_k_*}. A harmonic is counted if $A_{h}/N_{med}>\tau$ (*τ* = 1.5). Denote the number of matched harmonics *M*; define:

(30) $$\mathrm{HC}=\frac{M}{H}$$

where 0 ≤ HC ≤ 1. In experiments *H* is chosen according to the expected harmonic range.

#### 3.2.3. Indicator Normalization, Composite Score and Selection Rule

Because the five metrics have different dynamic ranges, each raw metric is min–max normalized across the IMFs of the same decomposition:

(31) $$X_{j}=\frac{X_{j}-{min}_{i}X_{i}}{{max}_{i}X_{i}-{min}_{i}X_{i}+ò}$$

where $X_{j}$ is the raw value for IMF *j* (X ∈ { SF, CF, *Kurt*(*E*), EC, HC} and *ϵ* prevents division by zero. After normalization all indicators lie in [0, 1] and higher values indicate stronger fault relevance.

(32) $$S_{j}=w_{sc}\left({\tilde{\mathrm{SF}}}_{j}+{\tilde{\mathrm{CF}}}_{j}\right)+w_{\mathrm{EPK}}{\tilde{\mathrm{EPK}}}_{j}+w_{\mathrm{EC}}{\tilde{\mathrm{EC}}}_{j}+w_{\mathrm{HC}}{\tilde{\mathrm{HC}}}_{j}$$

where ∑w = 1. In this work, weights were determined empirically by Pareto experiments and set as $w_{sc}$ = 0.125, $w_{\mathrm{EPK}}$ = 0.30, $w_{\mathrm{EC}}$ = 0.25, $w_{\mathrm{HC}}$ = 0.2.

The adaptive selection rule is:

(1) Rank IMFs by $S_{j}$ in descending order.
(2) Retain all IMFs with $S_{j}$ > *θ* (we use *θ* = 0.5 in experiments).
(3) If fewer than two IMFs satisfy $S_{j}$ > *θ*, retain the two highest-scoring IMFs to preserve sufficient fault information.
(4) Reconstruct the feature signal by linear superposition of the retained IMFs:
  (33) $$x_{\mathrm{recon}}\left(t\right)=\sum_{j\in S}\mathrm{IM}F_{j}\left(t\right)$$
  where *S* is the selected index set.

The reconstructed signal $x_{\mathrm{recon}}(t)$ is then subjected to envelope demodulation for final fault-frequency identification. All normalization, scoring and selection steps use the same spectral parameters (FFT length, window, overlap) to ensure comparability across methods.

## 4. Experimental Verification

### 4.1. Simulation Signal Analysis

A simulated inner-ring fault signal was generated to validate the effectiveness of the proposed ICPO–FMD framework under strong background noise. The simulated vibration follows a standard impulse-response model:

(34) $$y\left(t\right)=\sum_{k}A_{k}h\left(t-kT_{1}-\tau_{k}\right)+s\left(t\right)$$

(35) $$A_{k}=A_{0}\sin\left(2\pi f_{r}t\right)+1$$

(36) $$h\left(t\right)=\exp\left(-Ct\right)\sin\left(2\pi f_{n}t\right)$$

where *T*_1_ denotes the impulse repetition period, and *s*(*t*) denotes additive Gaussian white noise. Parameters used in the simulation are: Initial amplitude *A*_0_ = 0.5, the rotational frequency $f_{r}$ = 25 Hz, attenuation coefficient *C* = 800, the resonant frequency $f_{n}$ = 4000 Hz, the inner ring fault frequency $f_{i}$ = 120 Hz, and sampling frequency *f_s_* = 12.8 kHz. The signal-to-noise ratio (SNR) of *s*(*t*) is set to −12 dB to simulate adverse measurement conditions.

**Figure 3:**
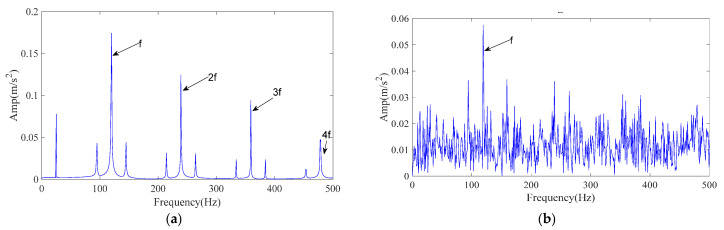
Simulated signal envelope spectrum. (**a**) The envelope spectrum of the simulated signal; (**b**) The envelope spectrum after adding noise.

Figure 3a shows the envelope spectrum of the clean simulated signal and Figure 3b shows the envelope spectrum after adding noise. As seen in Figure 3b, the 2–4× fault frequencies are significantly submerged by noise, which makes direct detection of the fault signature challenging.

**Figure 4:**
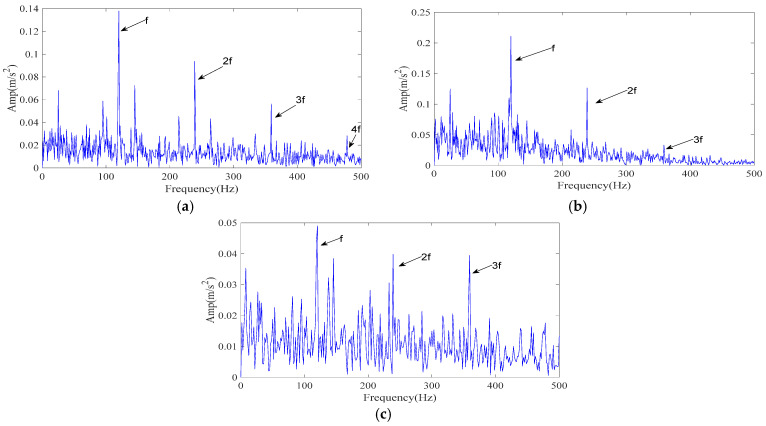
The envelope spectrum of the reconstructed signal resulting from different methods (simulated signal). (**a**) ICPO-FMD; (**b**) CPO-FMD; (**c**) ICPO-VMD.

The simulated signal was processed by the proposed ICPO–FMD procedure as follows. First, ICPO was employed to search the parameter space (*L*, *K*) by minimizing the envelope entropy. The optimization returned the optimal parameter set (*L*, *K*) = (40, 3). Using these parameters, FMD decomposed the noisy vibration into three IMFs. The IMF selection strategy detailed in Section 3.2 was then applied without modification to all methods for a fair comparison; and the IMFs selected by the scoring model were used to generate the reconstructed signals. The envelope spectrum of the ICPO–FMD reconstructed signal is presented in Figure 4a. After reconstruction, the 1–4× fault characteristic frequencies become clearly distinguishable, indicating that the proposed processing scheme can effectively recover weak periodic impulses masked by strong noise.

To evaluate the relative performance, two reference methods were tested under identical conditions: CPO–FMD and ICPO–VMD. Results are reported in Figure 4b,c. For ICPO–VMD (Figure 4c), although the 1–3× fault characteristic frequencies can be clearly detected, a considerable number of interference frequencies are also present.

The experimental results demonstrate that ICPO–FMD provides the most complete detection of fault characteristic frequencies (1–4×) and exhibits superior capability in recovering impulse features under a signal-to-noise ratio of −12 dB. This finding verifies that the joint optimization of parameters (*L*, *K*) by ICPO, in combination with the FMD and the proposed IMF selection scheme, can significantly enhance the extraction of weak periodic impulse signals.

In order to fully verify the effectiveness of the method proposed in this paper, this paper will conduct verification on the outer ring faults of the simulation signal and the ball faults of the simulation signal.

A simulated outer-ring fault signal was generated to validate the effectiveness of the proposed ICPO–FMD framework under strong background noise. The simulated vibration follows a standard impulse-response model:

(37) $$x\left(t\right)=\sum e^{-2\pi gtf_{n}}\cdot A_{0}\sin\left[2\pi f_{n}\sqrt{\left(1-g^{2}\right)}t\right]$$

(38) $$y\left(t\right)=x\left(t\right)+n\left(t\right)$$

**Figure 5:**
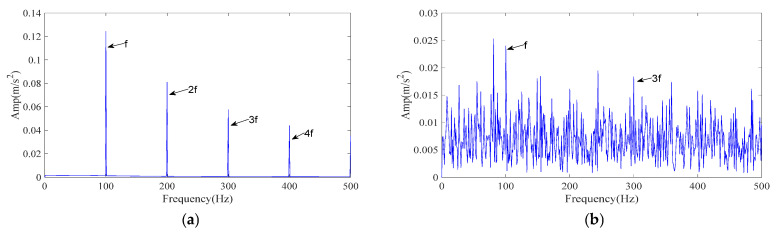
Simulated signal envelope spectrum. (**a**) The envelope spectrum of the simulated signal; (**b**) The envelope spectrum after adding noise.

In the formula, the natural frequency fn = 3000 Hz, the number of sampling points is 20,000; the sampling frequency is 20,000 Hz; the displacement constant A0 = 0.6; the damping coefficient g = 0.04; the period is 0.01 s; and n(t) is the added white noise. Figure 5a shows the envelope spectrum of the clean simulated signal and Figure 5b shows the envelope spectrum after adding noise. As seen in Figure 5b, the 1–4× fault frequencies are significantly submerged by noise, which makes direct detection of the fault signature challenging.

**Figure 6:**
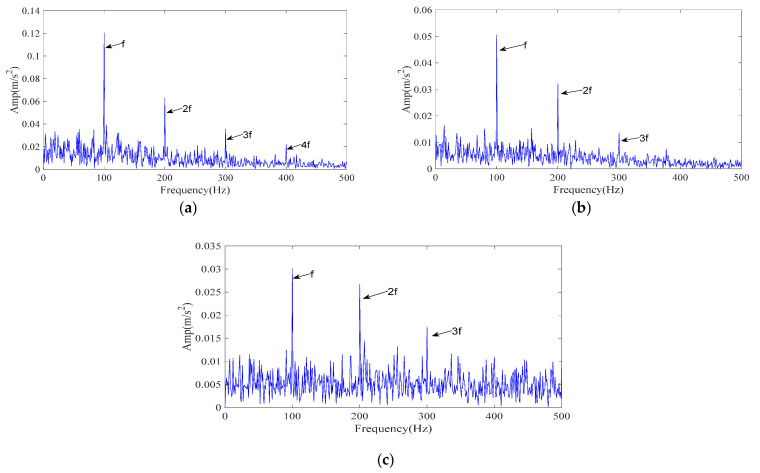
The envelope spectrum of the reconstructed signal resulting from different methods (simulated signal). (**a**) ICPO-FMD; (**b**) CPO-FMD; (**c**) ICPO-VMD.

The simulated signal was processed by the proposed ICPO–FMD procedure as follows. First, ICPO was employed to search the parameter space (*L*, *K*) by minimizing the envelope entropy. The optimization returned the optimal parameter set (*L*, *K*) = (49, 5). Using these parameters, FMD decomposed the noisy vibration into five IMFs. The IMF selection strategy detailed in Section 3.2 was then applied without modification to all methods for a fair comparison; and the IMFs selected by the scoring model were used to generate the reconstructed signals. The envelope spectrum of the ICPO–FMD reconstructed signal is presented in Figure 6a. After reconstruction, the 1–4× fault characteristic frequencies become clearly distinguishable, indicating that the proposed processing scheme can effectively recover weak periodic impulses masked by strong noise.

To evaluate the relative performance, two reference methods were tested under identical conditions: CPO–FMD and ICPO–VMD. Results are reported in Figure 6b,c. For ICPO–VMD (Figure 6c), although the 1–3× fault characteristic frequencies can be clearly detected, a considerable number of interference frequencies are also present.

The experimental results demonstrate that ICPO–FMD provides the most complete detection of fault characteristic frequencies (1–4×) and exhibits superior capability in recovering impulse features under a signal-to-noise ratio of –15 dB. This finding verifies that the joint optimization of parameters (*L*, *K*) by ICPO, in combination with the FMD and the proposed IMF selection scheme, can significantly enhance the extraction of weak periodic impulse signals.

### 4.2. Dataset Signal Analysis

**Figure 7:**
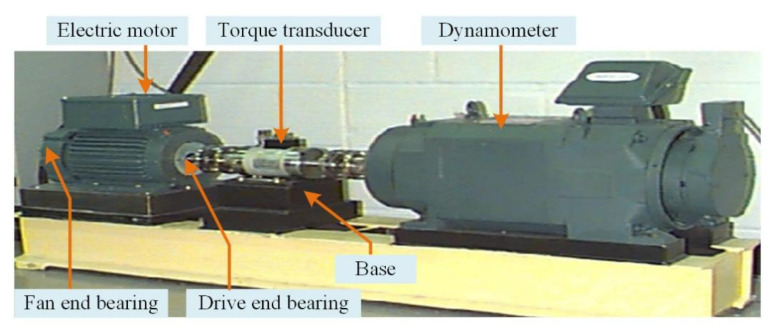
CWRU test rig.

The effectiveness of the proposed ICPO–FMD method was further validated on the Case Western Reserve University (CWRU) bearing test rig with a focus on inner-ring defects. The test bearings were mounted at the motor driving end and a single-point inner-ring defect was introduced by electrical discharge machining. Acceleration sensors were used for data acquisition at a sampling frequency of 12 kHz. Measurements were collected under four rotational speeds (1730, 1750, 1772 and 1797 r/min) for both healthy and faulty bearings. Figure 7 shows the test rig.

**Figure 8:**
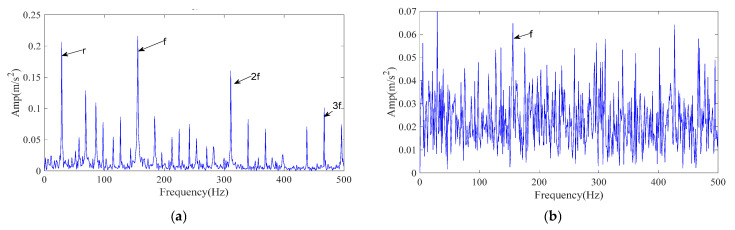
Envelope spectrum of CWRU test rig signals. (**a**) Envelope spectrum of CWRU raw signal; (**b**) Envelope spectrum of CWRU noised signal.

For demonstration, a 1-s segment of the inner-ring fault signal at 1730 r/min was processed. The envelope spectrum of the original signal is shown in Figure 8a, where impact-related components are visible. To evaluate robustness under severe interference, zero-mean Gaussian white noise was added to obtain an overall SNR of −9.88 dB; the noisy envelope spectrum is shown in Figure 8b, where fault-related harmonics are substantially masked by noise.

**Figure 9:**
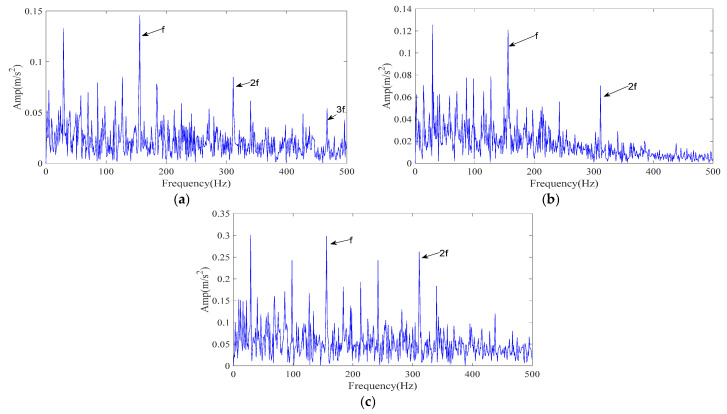
The envelope spectrum of the reconstructed signal resulting from different methods (CWRU signal). (**a**) ICPO-FMD; (**b**) CPO-FMD; (**c**) ICPO-VMD.

The processing procedure is described in Section 3. Specifically, the ICPO method is employed to optimize the FMD parameters (*L*, *K*) by minimizing the envelope entropy. Finally, the optimal parameter combination (42, 4) was obtained. obtained optimal parameter set is then applied to the noisy data for FMD analysis, resulting in four IMFs. Each IMF is evaluated and ranked using the time–frequency scoring model described in Section 3.2. According to the adaptive selection rule, IMF2 and IMF4 are retained for signal reconstruction. Figure 9a presents the envelope spectrum of the reconstructed signal. As shown, the 1–3× harmonics of the inner-ring fault characteristic frequency are clearly distinguishable, confirming that the proposed ICPO–FMD method can accurately identify inner-ring faults even at a noise level of −9.88 dB.

To verify the superiority of the proposed method, CPO–FMD and ICPO–VMD were selected as comparison methods and applied to the same noisy case with identical post-processing procedures. The experimental results of the comparison methods are presented in Figure 9b,c. As shown in Figure 9b, CPO–FMD could only recover the 1–2× fault characteristic frequencies, with the intensity of higher-order components being significantly weaker than that of ICPO–FMD. Similarly, ICPO–VMD was only able to identify the 1–2× fault characteristic frequencies and failed to recover the third harmonic component with comparable amplitude, as illustrated in Figure 9c. The experimental results based on the CWRU dataset further confirm the effectiveness and superiority of the proposed method.

The effectiveness of the proposed ICPO–FMD method was further validated on the Case Western Reserve University (CWRU) bearing test rig with a focus on outer-ring defects. The test bearings were mounted at the motor driving end and a single-point outer-ring defect was introduced by electrical discharge machining. Acceleration sensors were used for data acquisition at a sampling frequency of 12 kHz. Measurements were collected under four rotational speeds (1730, 1750, 1772 and 1797 r/min) for both healthy and faulty bearings.

**Figure 10:**
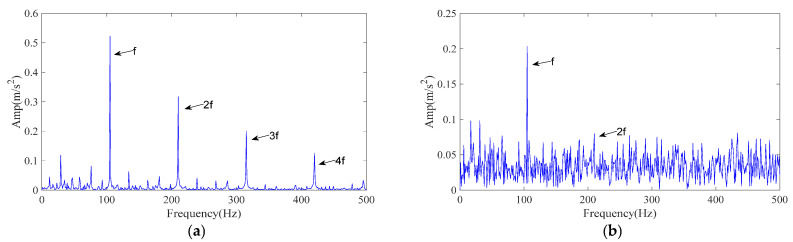
Envelope spectrum of CWRU test rig signals. (**a**) Envelope spectrum of CWRU raw signal; (**b**) Envelope spectrum of CWRU noised signal.

For demonstration, a 1-s segment of the outer-ring fault signal at 1772 r/min was processed. The envelope spectrum of the original signal is shown in Figure 10a, where impact-related components are visible. In order to verify the feature extraction capability of the proposed method in a strong noise interference environment, 7 dB Gaussian white noise was added to the original signal. The envelope spectrum of the noisy signal is shown in Figure 10b. Only a single harmonic can be clearly seen.

**Figure 11:**
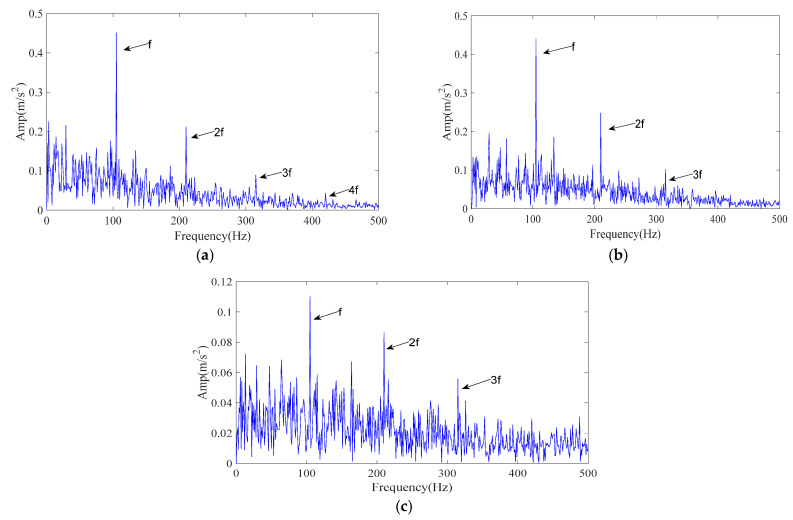
The envelope spectrum of the reconstructed signal resulting from different methods (CWRU signal). (**a**) ICPO-FMD; (**b**) CPO-FMD; (**c**) ICPO-VMD.

The processing procedure is described in Section 3. Specifically, the ICPO method is employed to optimize the FMD parameters (*L*, *K*) by minimizing the envelope entropy. Finally, the optimal parameter combination (46, 5) was obtained. The obtained optimal parameter set is then applied to the noisy data for FMD analysis, resulting in five IMFs. Each IMF is evaluated and ranked using the time–frequency scoring model described in Section 3.2. According to the adaptive selection rule, IMF2 and IMF3 are retained for signal reconstruction. Figure 11a presents the envelope spectrum of the reconstructed signal. As shown, the 1–4× harmonics of the outer-ring fault characteristic frequency are clearly distinguishable, confirming that the proposed ICPO–FMD method can accurately identify outer-ring faults.

To verify the superiority of the proposed method, CPO–FMD and ICPO–VMD were selected as comparison methods and applied to the same noisy case with identical post-processing procedures. The experimental results of the comparison methods are presented in Figure 11b,c. As shown in Figure 11b, CPO–FMD could only recover the 1–3× fault characteristic frequencies, with the intensity of higher-order components being significantly weaker than that of ICPO–FMD. Similarly, ICPO–VMD was only able to identify the 1–3× fault characteristic frequencies and failed to recover the third harmonic component with comparable amplitude, as illustrated in Figure 11c. The experimental results based on the CWRU dataset further confirm the effectiveness and superiority of the proposed method.

### 4.3. Nanchang Railway Bureau Signal Analysis

**Figure 12:**
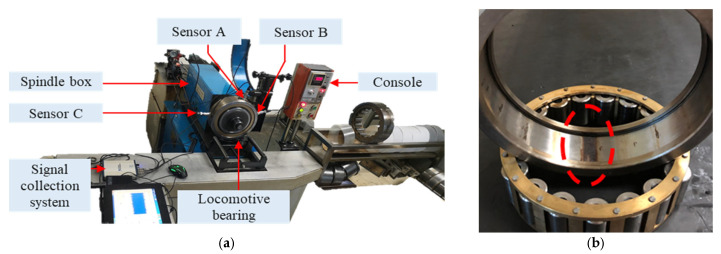
Test rig of Nanchang Railway Bureau. (**a**) JL-501 test rig; (**b**) Test bearing with outer ring fault.

To further validate the applicability of the proposed method to engineering signals, evaluations were conducted using engineering data from the JL-501 test rig at the Nanchang Railway Bureau locomotive depot. As shown in Figure 12a, the test rig consists of three sensors, a hydraulic system, a spindle box, a data acquisition system, a control console, and the tested bearing. The vibration data were collected at a sampling frequency of 20 kHz. The test bearing is the NJ2232WB cylindrical roller bearing produced by Wafangdian Tiema Railway Bearing Manufacturing Co., Ltd. in Dalian, China (inner diameter: 160 mm; outer diameter: 290 mm). In this study, an outer race fault was taken as an example to evaluate the proposed method, and the experimental bearing is illustrated in Figure 8b.

**Figure 13:**
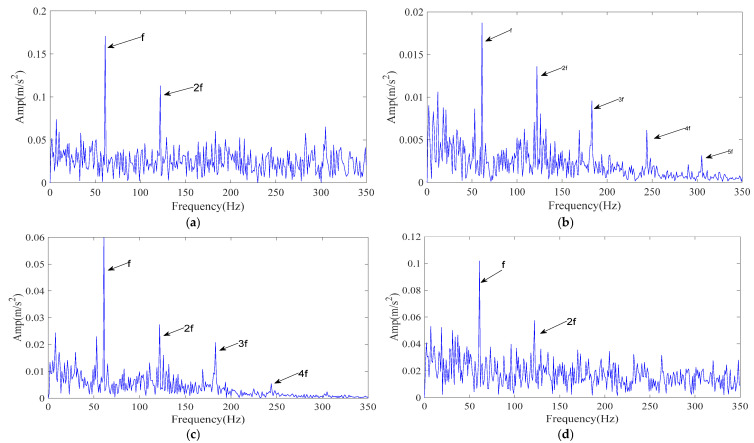
The envelope spectrum of the reconstructed signal with different methods. (**a**) Raw signal envelope spectrum; (**b**) ICPO-FMD; (**c**) CPO-FMD; (**d**) ICPO-VMD.

A one-second segment of the signal recorded by sensor B was selected for detailed analysis. Based on the calculation of the outer race fault characteristic frequency, the theoretical frequency at the experimental speed was determined to be 61 Hz. The envelope spectrum of the raw vibration data is presented in Figure 13a. As shown, although spectral lines can be observed at the 1× and 2× harmonics of the fault characteristic frequency, numerous interference frequencies are present, and the visibility of higher-order components is limited.

The processing procedure follows the workflow described in Section 3. First, the ICPO algorithm is employed to optimize the FMD parameters (*L*, *K*) by minimizing the envelope entropy of the reconstructed signal. Finally, the optimal parameter combination (23, 8) was obtained.

Based on the optimized parameters, FMD decomposition produces multiple IMFs, which are evaluated and filtered according to the time- and frequency-domain criteria described in Section 3.2. The retained IMFs are then recombined for envelope analysis. The resulting envelope spectrum, shown in Figure 13b, exhibits clear peaks at the 1–5× harmonics of the theoretical fault characteristic frequency, which dominate the entire spectrum. This demonstrates that the proposed method significantly enhances the harmonic structure associated with outer race faults and enables reliable fault feature identification under practical conditions.

For a controlled comparison, the same vibration signal was also processed using CPO–FMD and ICPO–VMD. The experimental results are shown in Figure 13c,d. As illustrated in Figure 13c, CPO–FMD mainly recovered the 1–4× fault characteristic frequencies but failed to restore higher-order harmonics with comparable amplitudes. In contrast, the envelope spectrum of the reconstructed signal obtained by ICPO–VMD revealed the 1–2× fault characteristic frequencies, yet their amplitudes were markedly lower than those achieved by ICPO–FMD, as shown in Figure 13d.

The superior performance of ICPO–FMD on engineering signals can be attributed to two main factors: (1) the multi-strategy search of ICPO generates parameter sets (*L*, *K*) that better match the time–frequency structure of the signal, thereby reducing mode mixing during the FMD process; and (2) the proposed IMF selection strategy retains useful components while suppressing noise-dominated IMFs. Consequently, ICPO–FMD can more completely recover fault-related harmonics under real operating conditions, thereby enhancing fault detectability.

## 5. Conclusions

This study proposes a rolling bearing fault diagnosis framework that combines the improved crested porcupine optimizer with feature modal decomposition. The ICPO algorithm is employed with the minimum envelope entropy as an adaptive optimization objective function to optimize the key parameters of FMD, namely the filter length *L* and the number of decomposition modes *K*. Additionally, a multi-criterion IMF selection strategy is developed to enhance the retention of fault-related components. Based on the findings, the following conclusions can be drawn:

(1) The optimization performance of ICPO was comprehensively validated on 29 benchmark functions from the CEC2017 test suite, in comparison with nine representative swarm intelligence algorithms, including CPO, TOC, SFOA, SABO, ZOA, DBO, SSA, and GJO. The results demonstrate that ICPO exhibits superior optimization capability, outperforming the original CPO algorithm in 26 out of 29 cases, thereby confirming the effectiveness of the proposed improvements.
(2) The proposed ICPO–FMD fault diagnosis framework was systematically validated using three types of signals: (i) simulated bearing vibration data, (ii) the CWRU experimental dataset, and (iii) engineering vibration signals acquired from the JL-501 test rig at Nanchang Locomotive Depot. Across all cases, the method can effectively optimized the FMD parameters, adaptively selected IMFs enriched with fault-related information, and reconstructed signals through envelope analysis that clearly revealed the fault characteristic frequencies.
(3) The proposed method demonstrates outstanding robustness under strong noise interference. By enhancing the clarity of harmonics and suppressing noise-dominated components, the ICPO–FMD technique effectively extracts fault features and their harmonics, enabling precise and reliable bearing fault diagnosis. These results highlight the significant potential of the proposed method for practical engineering applications.

In future research, we will extend the framework to non-stationary conditions via order tracking and online adaptive ICPO, integrate deep learning (CNNs/LSTMs) for automated diagnosis with interpretable attention mechanisms, and achieve sub-second real-time performance through incremental optimization and GPU/FPGA acceleration.

The proposed method has several limitations: (1) Hyperparameter sensitivity may cause premature convergence without self-adaptive tuning; (2) non-stationary adaptability requires adaptive windowing for time-varying fault frequencies; (3) computational cost (15–35 s per window) necessitates hierarchical optimization for industrial deployment; (4) applicability boundaries exist for compound faults or SNR < −25 dB, requiring preprocessing (spectral subtraction) or post-separation (ICA); and (5) cross-domain generalization across bearing types and machinery (gearboxes, roller bearings) needs systematic validation. Addressing these through cross-dataset evaluation is crucial for transitioning ICPO-FMD to industrial-scale predictive maintenance.

## Data Availability

The original contributions presented in this study are included in the article. Further inquiries can be directed to the corresponding author.

## Appendix A. CEC2017 Optimization Function Test Set

**Table A1:** CEC2017 Optimization Function Test Set.

	No	Functions	*F_i_* = F_i_(x)
Unimodal	1	Shifted and Rotated Bent Cigar Function	100
Functions	3	Shifted and Rotated Zakharov Function	300
Simple Multimodal Functions Hybrid Functions Composition Functions	4 5 6 7 8 9 10 11 12 13 14 15 16 17 18 19 20 21 22 23 24 25 26 27 28 29 30	Shifted and Rotated Rosenbrock’s Function Shifted and Rotated Rastrigin’s Function Shifted and Rotated ExLiuded Scaffer’s F6 Function Shifted and Rotated Lunacek Bi_Rastrigin Function Shifted and Rotated Non-Continuous Rastrigin’s Function Shifted and Rotated Levy Function Shifted and Rotated Schwefel’s Function Hybrid Function 1 (*N* = 3) Hybrid Function 2 (*N* = 3) Hybrid Function 3 (*N* = 3) Hybrid Function 4 (*N* = 4) Hybrid Function 5 (*N* = 4) Hybrid Function 6 (*N* = 4) Hybrid Function 6 (*N* = 5) Hybrid Function 6 (*N* = 5) Hybrid Function 6 (*N* = 5) Hybrid Function 6 (*N* = 6) Composition Function 1 (*N* = 3) Composition Function 2 (*N* = 3) Composition Function3 (*N* = 4) Composition Function 4 (*N* = 4) Composition Function 5 (*N* = 5) Composition Function 6 (*N* = 5) Composition Function 7 (*N* = 6) Composition Function 8 (*N* = 6) Composition Function 9 (*N* = 3) Composition Function 10 (*N* = 3)	400 500 600 700 800 900 1000 1100 1200 1300 1400 1500 1600 1700 1800 1900 2000 2100 2200 2300 2400 2500 2600 2700 2800 2900 3000

## Appendix B. Indicator Results

**Table A2:** Indicator Results.

Function	Result	ICPO	CPO	TOC	SFOA	SABO	ZOA	DBO	SSA	GJO
F1	Best	1.08 × 10^2^	1.88 × 10^7^	9.10 × 10^9^	3.49 × 10^10^	7.08 × 10^10^	5.35 × 10^10^	2.43 × 10^8^	5.44 × 10^6^	9.20 × 10^10^
	Std	3.34 × 10^3^	8.08 × 10^6^	1.40 × 10^10^	9.15 × 10^9^	1.25 × 10^10^	1.37 × 10^10^	2.13 × 10^9^	3.25 × 10^6^	1.12 × 10^10^
	Avg	3.09 × 10^3^	2.96 × 10^7^	2.03 × 10^10^	5.21 × 10^10^	9.09 × 10^10^	8.60 × 10^10^	3.13 × 10^9^	1.00 × 10^7^	1.13 × 10^11^
	P		3.02 × 10^−11^	3.02 × 10^−11^	3.02 × 10^−11^	3.02 × 10^−11^	3.02 × 10^−11^	3.02 × 10^−11^	3.02 × 10^−11^	3.02 × 10^−11^
	Rank	1	3	5	6	8	7	4	2	9
F3	Best	1.88 × 10^5^	3.14 × 10^5^	2.25 × 10^5^	1.67 × 10^5^	3.02 × 10^5^	2.39 × 10^5^	3.62 × 10^5^	3.48 × 10^5^	2.37 × 10^5^
	Std	2.32 × 10^4^	2.84 × 10^4^	2.35 × 10^5^	4.83 × 10^4^	1.17 × 10^4^	1.43 × 10^4^	2.11 × 10^5^	6.14 × 10^4^	1.85 × 10^4^
	Avg	2.35 × 10^5^	3.66 × 10^5^	4.50 × 10^5^	2.24 × 10^5^	3.31 × 10^5^	2.68 × 10^5^	6.16 × 10^5^	4.43 × 10^5^	2.77 × 10^5^
	P		3.02 × 10^−11^	4.69 × 10^−8^	6.14 × 10^−2^	3.02 × 10^−11^	2.78 × 10^−7^	3.02 × 10^−11^	3.02 × 10^−11^	1.31 × 10^−8^
	Rank	2	6	7	1	5	3	9	8	4
F4	Best	5.56 × 10^2^	8.33 × 10^2^	2.06 × 10^3^	3.73 × 10^3^	7.13 × 10^3^	6.14 × 10^3^	9.61 × 10^2^	7.49 × 10^2^	8.62 × 10^3^
	Std	4.72 × 10^1^	4.16 × 10^1^	1.09 × 10^3^	1.22 × 10^3^	3.98 × 10^3^	3.59 × 10^3^	2.45 × 10^2^	6.29 × 10^1^	3.37 × 10^3^
	Avg	6.74 × 10^2^	8.97 × 10^2^	3.47 × 10^3^	5.87 × 10^3^	1.45 × 10^4^	1.07 × 10^4^	1.35 × 10^3^	8.57 × 10^2^	1.30 × 10^4^
	P		3.02 × 10^−11^	3.02 × 10^−11^	3.02 × 10^−11^	3.02 × 10^−11^	3.02 × 10^−11^	3.02 × 10^−11^	3.69 × 10^−11^	3.02 × 10^−11^
	Rank	1	3	5	6	9	7	4	2	8
F5	Best	7.75 × 10^2^	1.42 × 10^3^	1.36 × 10^3^	1.77 × 10^3^	1.56 × 10^3^	1.29 × 10^3^	1.38 × 10^3^	1.32 × 10^3^	1.36 × 10^3^
	Std	9.17 × 10^1^	3.07 × 10^1^	1.25 × 10^2^	6.55 × 10^1^	8.84 × 10^1^	6.76 × 10^1^	1.51 × 10^2^	3.31 × 10^1^	8.74 × 10^1^
	Avg	9.26 × 10^2^	1.48 × 10^3^	1.72 × 10^3^	1.89 × 10^3^	1.81 × 10^3^	1.39 × 10^3^	1.68 × 10^3^	1.38 × 10^3^	1.51 × 10^3^
	P		3.02 × 10^−11^	3.02 × 10^−11^	3.02 × 10^−11^	3.02 × 10^−11^	3.02 × 10^−11^	3.02 × 10^−11^	3.02 × 10^−11^	3.02 × 10^−11^
	Rank	1	4	7	9	8	3	6	2	5
F6	Best	6.03 × 10^2^	6.03 × 10^2^	6.72 × 10^2^	6.82 × 10^2^	6.65 × 10^2^	6.60 × 10^2^	6.49 × 10^2^	6.63 × 10^2^	6.58 × 10^2^
	Std	6.30	9.80 × 10^−1^	6.80	6.70	1.13 × 10^1^	4.50	9.30	2.00	6.20
	Avg	6.12 × 10^2^	6.06 × 10^2^	6.87 × 10^2^	6.98 × 10^2^	6.84 × 10^2^	6.72 × 10^2^	6.70 × 10^2^	6.66 × 10^2^	6.67 × 10^2^
	P		2.38 × 10^−7^	3.02 × 10^−11^	3.02 × 10^−11^	3.02 × 10^−11^	3.02 × 10^−11^	3.02 × 10^−11^	3.02 × 10^−11^	3.02 × 10^−11^
	Rank	2	1	8	9	7	6	5	3	4
F7	Best	1.10 × 10^3^	1.74 × 10^3^	3.28 × 10^3^	2.50 × 10^3^	2.62 × 10^3^	2.79 × 10^3^	1.68 × 10^3^	2.93 × 10^3^	2.42 × 10^3^
	Std	9.99 × 10^1^	5.62 × 10^1^	3.75 × 10^2^	1.69 × 10^2^	1.45 × 10^2^	1.35 × 10^2^	2.70 × 10^2^	1.25 × 10^2^	1.54 × 10^2^
	Avg	1.24 × 10^3^	1.82 × 10^3^	3.86 × 10^3^	2.87 × 10^3^	2.84 × 10^3^	3.06 × 10^3^	2.25 × 10^3^	3.20 × 10^3^	2.76 × 10^3^
	P		3.02 × 10^−11^	3.02 × 10^−11^	3.02 × 10^−11^	3.02 × 10^−11^	3.02 × 10^−11^	3.02 × 10^−11^	3.02 × 10^−11^	3.02 × 10^−11^
	Rank	1	2	9	6	5	7	3	8	4
F8	Best	1.10 × 10^3^	1.71 × 10^3^	1.93 × 10^3^	2.08 × 10^3^	1.81 × 10^3^	1.65 × 10^3^	1.62 × 10^3^	1.61 × 10^3^	1.69 × 10^3^
	Std	7.78 × 10^1^	3.71 × 10^1^	1.03 × 10^2^	6.97 × 10^1^	1.17 × 10^2^	6.70 × 10^1^	1.85 × 10^2^	6.26 × 10^1^	1.14 × 10^2^
	Avg	1.19 × 10^3^	1.78 × 10^3^	2.12 × 10^3^	2.25 × 10^3^	2.09 × 10^3^	1.80 × 10^3^	2.00 × 10^3^	1.85 × 10^3^	1.85 × 10^3^
	P		3.02 × 10^−11^	3.02 × 10^−11^	3.02 × 10^−11^	3.02 × 10^−11^	3.02 × 10^−11^	3.02 × 10^−11^	3.02 × 10^−11^	3.02 × 10^−11^
	Rank	1	2	8	9	7	3	6	4	5
F9	Best	2.99 × 10^3^	7.67 × 10^3^	2.37 × 10^4^	6.47 × 10^4^	3.54 × 10^4^	2.89 × 10^4^	2.18 × 10^4^	2.36 × 10^4^	2.52 × 10^4^
	Std	7.00 × 10^3^	6.10 × 10^3^	1.18 × 10^4^	7.01 × 10^4^	1.28 × 10^4^	3.91 × 10^3^	1.83 × 10^4^	1.10 × 10^3^	1.27 × 10^4^
	Avg	9.68 × 10^3^	1.62 × 10^4^	4.28 × 10^4^	7.86 × 10^4^	6.27 × 10^4^	3.80 × 10^4^	4.61 × 10^4^	2.43 × 10^4^	4.96 × 10^4^
	P		5.86 × 10^−6^	9.92 × 10^−11^	3.02 × 10^−11^	4.08 × 10^−11^	1.78 × 10^−10^	1.21 × 10^−10^	6.52 × 10^−9^	6.07 × 10^−11^
	Rank	1	2	5	9	8	4	6	3	7
F10	Best	1.22 × 10^4^	2.77 × 10^4^	2.19 × 10^4^	2.99 × 10^4^	3.09 × 10^4^	1.74 × 10^4^	1.40 × 10^4^	1.50 × 10^4^	1.83 × 10^4^
	Std	5.86 × 10^3^	5.32 × 10^2^	2.48 × 10^3^	8.51 × 10^2^	5.06 × 10^2^	1.30 × 10^3^	4.37 × 10^3^	1.42 × 10^3^	5.29 × 10^3^
	Avg	2.30 × 10^4^	2.90 × 10^4^	2.70 × 10^4^	3.18 × 10^4^	3.20 × 10^4^	1.99 × 10^4^	1.88 × 10^4^	1.72 × 10^4^	2.38 × 10^4^
	P		7.69 × 10^−8^	1.50 × 10^−2^	3.02 × 10^−11^	3.02 × 10^−11^	6.37 × 10^−3^	6.09 × 10^−3^	3.01 × 10^−4^	8.90 × 10^−1^
	Rank	4	7	6	8	9	3	2	1	5
F11	Best	2.04 × 10^3^	2.07 × 10^4^	7.83 × 10^3^	9.47 × 10^3^	1.23 × 10^5^	2.69 × 10^4^	5.18 × 10^4^	1.47 × 10^4^	5.50 × 10^4^
	Std	1.79 × 10^2^	6.63 × 10^3^	2.50 × 10^4^	1.03 × 10^4^	1.84 × 10^4^	1.50 × 10^4^	2.99 × 10^4^	1.05 × 10^4^	1.43 × 10^4^
	Avg	2.31 × 10^3^	3.21 × 10^4^	2.96 × 10^4^	2.09 × 10^4^	1.58 × 10^5^	5.06 × 10^4^	9.30 × 10^4^	2.85 × 10^4^	7.66 × 10^4^
	P		3.02 × 10^−11^	3.02 × 10^−11^	3.02 × 10^−11^	3.02 × 10^−11^	3.02 × 10^−11^	3.02 × 10^−11^	3.02 × 10^−11^	3.02 × 10^−11^
	Rank	1	5	4	2	9	6	8	3	7
F12	Best	5.99 × 10^5^	1.64 × 10^7^	1.44 × 10^9^	1.70 × 10^9^	6.24 × 10^9^	1.05 × 10^10^	5.14 × 10^8^	2.41 × 10^7^	1.66 × 10^10^
	Std	9.97 × 10^5^	9.33 × 10^6^	1.89 × 10^9^	2.49 × 10^9^	6.93 × 10^9^	9.91 × 10^9^	8.77 × 10^8^	3.81 × 10^7^	8.03 × 10^9^
	Avg	1.98 × 10^6^	3.40 × 10^7^	3.79 × 10^9^	5.59 × 10^9^	1.72 × 10^10^	2.57 × 10^10^	1.75 × 10^9^	8.20 × 10^7^	3.20 × 10^10^
	P		3.02 × 10^−11^	3.02 × 10^−11^	3.02 × 10^−11^	3.02 × 10^−11^	3.02 × 10^−11^	3.02 × 10^−11^	3.02 × 10^−11^	3.02 × 10^−11^
	Rank	1	2	5	6	7	8	4	3	9
F13	Best	1.56 × 10^3^	2.72 × 10^3^	9.15 × 10^6^	2.41 × 10^7^	5.58 × 10^8^	6.43 × 10^5^	1.18 × 10^5^	1.31 × 10^4^	3.19 × 10^9^
	Std	2.65 × 10^3^	1.94 × 10^3^	3.97 × 10^8^	5.23 × 10^8^	1.06 × 10^9^	2.44 × 10^9^	1.27 × 10^8^	9.36 × 10^3^	2.39 × 10^9^
	Avg	4.52 × 10^3^	5.65 × 10^3^	1.67 × 10^8^	2.27 × 10^8^	1.97 × 10^9^	3.86 × 10^9^	1.09 × 10^8^	3.05 × 10^4^	6.36 × 10^9^
	P		1.76 × 10^−2^	3.02 × 10^−11^	3.02 × 10^−11^	3.02 × 10^−11^	3.02 × 10^−11^	3.02 × 10^−11^	3.02 × 10^−11^	3.02 × 10^−11^
	Rank	1	2	5	6	7	8	4	3	9
F14	Best	7.14 × 10^4^	3.03 × 10^5^	1.57 × 10^5^	4.60 × 10^3^	9.91 × 10^6^	1.75 × 10^6^	6.97 × 10^5^	2.55 × 10^5^	3.79 × 10^6^
	Std	1.39 × 10^5^	3.21 × 10^5^	2.35 × 10^6^	1.21 × 10^5^	3.49 × 10^6^	2.83 × 10^6^	7.24 × 10^6^	5.59 × 10^5^	5.17 × 10^6^
	Avg	2.67 × 10^5^	6.91 × 10^5^	1.18 × 10^6^	8.21 × 10^4^	1.65 × 10^7^	5.86 × 10^6^	9.88 × 10^6^	1.11 × 10^6^	1.10 × 10^7^
	P		3.82 × 10^−9^	4.22 × 10^−4^	3.96 × 10^−8^	3.02 × 10^−11^	3.02 × 10^−11^	3.02 × 10^−11^	4.20 × 10^−10^	3.02 × 10^−11^
	Rank	2	3	5	1	9	6	7	4	8
F15	Best	1.63 × 10^3^	2.14 × 10^3^	1.20 × 10^5^	5.40 × 10^5^	7.96 × 10^7^	3.47 × 10^6^	9.48 × 10^4^	4.29 × 10^3^	1.14 × 10^8^
	Std	1.77 × 10^3^	6.85 × 10^2^	2.53 × 10^8^	1.87 × 10^6^	1.35 × 10^8^	9.12 × 10^8^	4.44 × 10^7^	6.46 × 10^3^	1.73 × 10^9^
	Avg	2.95 × 10^3^	2.84 × 10^3^	8.70 × 10^7^	1.94 × 10^6^	3.43 × 10^8^	8.05 × 10^8^	1.95 × 10^7^	1.14 × 10^4^	1.76 × 10^9^
	P		1.02 × 10^−1^	3.02 × 10^−11^	3.02 × 10^−11^	3.02 × 10^−11^	3.02 × 10^−11^	3.02 × 10^−11^	8.10 × 10^−10^	3.02 × 10^−11^
	Rank	2	1	6	4	7	8	5	3	9
F16	Best	4.08 × 10^3^	7.59 × 10^3^	7.19 × 10^3^	9.15 × 10^3^	1.01 × 10^4^	6.59 × 10^3^	7.10 × 10^3^	5.21 × 10^3^	7.01 × 10^3^
	Std	6.84 × 10^2^	5.85 × 10^2^	1.82 × 10^3^	8.81 × 10^2^	9.38 × 10^2^	8.79 × 10^2^	7.65 × 10^2^	6.45 × 10^2^	1.13 × 10^3^
	Avg	5.30 × 10^3^	9.03 × 10^3^	9.77 × 10^3^	1.13 × 10^4^	1.19 × 10^4^	8.67 × 10^3^	8.33 × 10^3^	6.39 × 10^3^	8.52 × 10^3^
	P		3.02 × 10^−11^	3.02 × 10^−11^	3.02 × 10^−11^	3.02 × 10^−11^	4.08 × 10^−11^	3.02 × 10^−11^	7.04 × 10^−7^	3.02 × 10^−11^
	Rank	1	6	7	8	9	5	3	2	4
F17	Best	3.36 × 10^3^	5.51 × 10^3^	5.97 × 10^3^	6.59 × 10^3^	6.92 × 10^3^	5.51 × 10^3^	6.43 × 10^3^	4.51 × 10^3^	5.12 × 10^3^
	Std	5.10 × 10^2^	2.93 × 10^2^	2.04 × 10^3^	7.30 × 10^2^	2.17 × 10^3^	3.81 × 10^3^	1.06 × 10^3^	5.91 × 10^2^	6.76 × 10^3^
	Avg	4.41 × 10^3^	6.16 × 10^3^	8.43 × 10^3^	8.14 × 10^3^	9.18 × 10^3^	1.04 × 10^4^	8.14 × 10^3^	5.86 × 10^3^	9.43 × 10^3^
	P		3.02 × 10^−11^	3.02 × 10^−11^	3.02 × 10^−11^	3.02 × 10^−11^	3.02 × 10^−11^	3.02 × 10^−11^	2.61 × 10^−10^	4.08 × 10^−11^
	Rank	1	3	6	4	7	9	5	2	8
F18	Best	1.80 × 10^5^	3.98 × 10^5^	1.77 × 10^5^	1.07 × 10^5^	6.59 × 10^6^	1.57 × 10^6^	6.31 × 10^5^	7.94 × 10^5^	3.15 × 10^6^
	Std	4.10 × 10^5^	6.94 × 10^5^	9.92 × 10^6^	6.55 × 10^5^	7.15 × 10^6^	2.36 × 10^6^	9.22 × 10^6^	9.15 × 10^5^	5.28 × 10^6^
	Avg	7.90 × 10^5^	1.57 × 10^6^	3.10 × 10^6^	6.17 × 10^5^	1.76 × 10^7^	4.82 × 10^6^	1.25 × 10^7^	1.88 × 10^6^	1.03 × 10^7^
	P		8.20 × 10^−7^	1.45 × 10^−1^	4.22 × 10^−3^	3.02 × 10^−11^	6.07 × 10^−11^	3.47 × 10^−10^	8.35 × 10^−8^	3.02 × 10^−11^
	Rank	2	3	5	1	9	6	8	4	7
F19	Best	1.98 × 10^3^	2.13 × 10^3^	1.86 × 10^6^	2.43 × 10^6^	9.54 × 10^7^	2.27 × 10^7^	6.78 × 10^5^	3.73 × 10^3^	1.38 × 10^8^
	Std	4.13 × 10^3^	1.93 × 10^3^	1.40 × 10^7^	1.01 × 10^9^	2.41 × 10^8^	8.67 × 10^8^	1.23 × 10^7^	7.77 × 10^3^	6.40 × 10^8^
	Avg	4.58 × 10^3^	3.69 × 10^3^	1.79 × 10^7^	1.94 × 10^8^	4.31 × 10^8^	6.57 × 10^8^	1.78 × 10^7^	1.17 × 10^4^	9.69 × 10^8^
	P		8.18 × 10^−1^	3.02 × 10^−11^	3.02 × 10^−11^	3.02 × 10^−11^	3.02 × 10^−11^	3.02 × 10^−11^	1.61 × 10^−6^	3.02 × 10^−11^
	Rank	2	1	5	6	7	8	4	3	9
F20	Best	3.71 × 10^3^	6.15 × 10^3^	4.87 × 10^3^	7.10 × 10^3^	7.01 × 10^3^	4.47 × 10^3^	4.86 × 10^3^	4.69 × 10^3^	4.53 × 10^3^
	Std	7.36 × 10^2^	2.28 × 10^2^	6.87 × 10^2^	3.09 × 10^2^	2.27 × 10^2^	2.87 × 10^2^	4.91 × 10^2^	6.42 × 10^2^	1.03 × 10^2^
	Avg	5.01 × 10^3^	6.69 × 10^3^	6.17 × 10^3^	7.66 × 10^3^	7.68 × 10^3^	4.91 × 10^3^	5.97 × 10^3^	5.92 × 10^3^	6.17 × 10^3^
	P		5.49 × 10^−11^	7.60 × 10^−7^	3.02 × 10^−11^	3.02 × 10^−11^	9.46 × 10^−1^	3.57 × 10^−6^	3.83 × 10^−5^	3.59 × 10^−5^
	Rank	2	7	5	8	9	1	4	3	6
F21	Best	2.57 × 10^3^	3.19 × 10^3^	3.69 × 10^3^	3.59 × 10^3^	3.85 × 10^3^	3.41 × 10^3^	3.41 × 10^3^	3.20 × 10^3^	3.17 × 10^3^
	Std	5.98 × 10^1^	2.22 × 10^1^	1.82 × 10^2^	1.12 × 10^2^	1.04 × 10^2^	1.24 × 10^2^	1.11 × 10^2^	2.29 × 10^2^	1.42 × 10^2^
	Avg	2.66 × 10^3^	3.24 × 10^3^	4.04 × 10^3^	3.82 × 10^3^	4.05 × 10^3^	3.69 × 10^3^	3.61 × 10^3^	3.71 × 10^3^	3.38 × 10^3^
	P		3.02 × 10^−11^	3.02 × 10^−11^	3.02 × 10^−11^	3.02 × 10^−11^	3.02 × 10^−11^	3.02 × 10^−11^	3.02 × 10^−11^	3.02 × 10^−11^
	Rank	1	2	8	7	9	5	4	6	3
F22	Best	1.60 × 10^4^	3.03 × 10^4^	2.55 × 10^4^	1.14 × 10^4^	2.37 × 10^4^	2.06 × 10^4^	1.78 × 10^4^	1.77 × 10^4^	2.06 × 10^4^
	Std	5.20 × 10^3^	5.16 × 10^2^	1.72 × 10^3^	4.95 × 10^3^	2.41 × 10^3^	1.42 × 10^3^	2.37 × 10^3^	1.20 × 10^3^	5.05 × 10^3^
	Avg	2.42 × 10^4^	3.14 × 10^4^	2.94 × 10^4^	3.19 × 10^4^	3.30 × 10^4^	2.33 × 10^4^	2.11 × 10^4^	2.01 × 10^4^	2.70 × 10^4^
	P		8.89 × 10^−10^	2.25 × 10^−4^	9.26 × 10^−9^	5.57 × 10^−10^	3.90 × 10^−1^	6.56 × 10^−2^	1.83 × 10^−2^	1.05 × 10^−1^
	Rank	4	7	6	8	9	3	2	1	5
F23	Best	3.08 × 10^3^	3.71 × 10^3^	4.73 × 10^3^	4.06 × 10^3^	4.65 × 10^3^	4.92 × 10^3^	3.94 × 10^3^	3.83 × 10^3^	3.93 × 10^3^
	Std	6.47 × 10^1^	3.46 × 10^1^	3.19 × 10^2^	1.21 × 10^2^	1.99 × 10^2^	2.92 × 10^2^	1.35 × 10^2^	1.90 × 10^2^	1.08 × 10^2^
	Avg	3.22 × 10^3^	3.78 × 10^3^	5.17 × 10^3^	4.29 × 10^3^	4.96 × 10^3^	5.49 × 10^3^	4.22 × 10^3^	4.21 × 10^3^	4.13 × 10^3^
	P		3.02 × 10^−11^	3.02 × 10^−11^	3.02 × 10^−11^	3.02 × 10^−11^	3.02 × 10^−11^	3.02 × 10^−11^	3.02 × 10^−11^	3.02 × 10^−11^
	Rank	1	2	8	6	7	9	4	5	3
F24	Best	3.59 × 10^3^	4.13 × 10^3^	5.56 × 10^3^	4.57 × 10^3^	5.56 × 10^3^	6.67 × 10^3^	4.70 × 10^3^	4.41 × 10^3^	4.99 × 10^3^
	Std	9.22 × 10^1^	4.01 × 10^1^	4.91 × 10^2^	2.22 × 10^2^	3.46 × 10^2^	3.37 × 10^2^	2.44 × 10^2^	3.54 × 10^2^	2.95 × 10^2^
	Avg	3.72 × 10^3^	4.21 × 10^3^	6.43 × 10^3^	5.01 × 10^3^	6.07 × 10^3^	7.22 × 10^3^	5.15 × 10^3^	5.13 × 10^3^	5.36 × 10^3^
	P		3.02 × 10^−11^	3.02 × 10^−11^	3.02 × 10^−11^	3.02 × 10^−11^	3.02 × 10^−11^	3.02 × 10^−11^	3.02 × 10^−11^	3.02 × 10^−11^
	Rank	1	2	8	3	7	9	5	4	6
F25	Best	3.18 × 10^3^	3.46 × 10^3^	4.48 × 10^3^	5.67 × 10^3^	7.39 × 10^3^	6.50 × 10^3^	3.58 × 10^3^	3.37 × 10^3^	7.91 × 10^3^
	Std	5.37 × 10^1^	4.65 × 10^1^	1.07 × 10^3^	8.22 × 10^2^	1.38 × 10^3^	1.51 × 10^3^	2.84 × 10^2^	7.33 × 10^1^	1.82 × 10^3^
	Avg	3.32 × 10^3^	3.59 × 10^3^	5.93 × 10^3^	6.99 × 10^3^	1.08 × 10^4^	8.47 × 10^3^	4.00 × 10^3^	3.51 × 10^3^	1.04 × 10^4^
	P		3.02 × 10^−11^	3.02 × 10^−11^	3.02 × 10^−11^	3.02 × 10^−11^	3.02 × 10^−11^	3.02 × 10^−11^	7.39 × 10^−11^	3.02 × 10^−11^
	Rank	1	3	5	6	9	7	4	2	8
F26	Best	2.90 × 10^3^	1.55 × 10^4^	2.70 × 10^4^	1.12 × 10^4^	3.14 × 10^4^	2.81 × 10^4^	1.81 × 10^4^	5.36 × 10^3^	1.86 × 10^4^
	Std	4.39 × 10^3^	7.31 × 10^2^	4.85 × 10^3^	3.86 × 10^3^	2.31 × 10^3^	2.41 × 10^3^	2.49 × 10^3^	4.47 × 10^3^	1.93 × 10^3^
	Avg	1.04 × 10^4^	1.64 × 10^4^	3.57 × 10^4^	2.08 × 10^4^	3.50 × 10^4^	3.36 × 10^4^	2.36 × 10^4^	2.45 × 10^4^	2.36 × 10^4^
	P		3.65 × 10^−8^	3.02 × 10^−11^	2.44 × 10^−9^	3.02 × 10^−11^	3.02 × 10^−11^	3.02 × 10^−11^	3.16 × 10^−10^	3.02 × 10^−11^
	Rank	1	2	9	3	8	7	4	6	5
F27	Best	3.46 × 10^3^	3.60 × 10^3^	4.15 × 10^3^	3.60 × 10^3^	4.69 × 10^3^	6.68 × 10^3^	3.76 × 10^3^	3.60 × 10^3^	4.26 × 10^3^
	Std	6.60 × 10^1^	3.95 × 10^1^	5.52 × 10^2^	9.87 × 10^1^	4.40 × 10^2^	7.71 × 10^2^	2.09 × 10^2^	2.31 × 10^2^	3.76 × 10^2^
	Avg	3.58 × 10^3^	3.66 × 10^3^	4.99 × 10^3^	3.75 × 10^3^	5.43 × 10^3^	8.32 × 10^3^	4.06 × 10^3^	3.95 × 10^3^	5.04 × 10^3^
	P		1.49 × 10^−6^	3.02 × 10^−11^	2.44 × 10^−9^	3.02 × 10^−11^	3.02 × 10^−11^	3.02 × 10^−11^	6.72 × 10^−10^	3.02 × 10^−11^
	Rank	1	2	6	3	8	9	5	4	7
F28	Best	3.41 × 10^3^	3.69 × 10^3^	4.43 × 10^3^	6.12 × 10^3^	1.21 × 10^4^	9.34 × 10^3^	3.72 × 10^3^	3.51 × 10^3^	1.06 × 10^4^
	Std	2.47 × 10^1^	4.02 × 10^1^	2.92 × 10^3^	1.24 × 10^3^	1.46 × 10^3^	1.73 × 10^3^	6.70 × 10^3^	4.54 × 10^1^	1.60 × 10^3^
	Avg	3.45 × 10^3^	3.77 × 10^3^	7.52 × 10^3^	8.54 × 10^3^	1.52 × 10^4^	1.22 × 10^4^	1.60 × 10^4^	3.60 × 10^3^	1.40 × 10^4^
	P		3.02 × 10^−11^	3.02 × 10^−11^	3.02 × 10^−11^	3.02 × 10^−11^	3.02 × 10^−11^	3.02 × 10^−11^	3.69 × 10^−11^	3.02 × 10^−11^
	Rank	1	3	4	5	8	6	9	2	7
F29	Best	5.17 × 10^3^	8.55 × 10^3^	1.10 × 10^4^	9.88 × 10^3^	1.30 × 10^4^	1.00 × 10^4^	8.34 × 10^3^	7.03 × 10^3^	9.70 × 10^3^
	Std	4.35 × 10^2^	2.67 × 10^2^	2.34 × 10^3^	8.75 × 10^2^	2.31 × 10^3^	1.56 × 10^3^	1.31 × 10^3^	5.49 × 10^2^	1.94 × 10^3^
	Avg	6.24 × 10^3^	9.12 × 10^3^	1.60 × 10^4^	1.15 × 10^4^	1.69 × 10^4^	1.37 × 10^4^	1.01 × 10^4^	8.07 × 10^3^	1.26 × 10^4^
	P		3.02 × 10^−11^	3.02 × 10^−11^	3.02 × 10^−11^	3.02 × 10^−11^	3.02 × 10^−11^	3.02 × 10^−11^	3.69 × 10^−11^	3.02 × 10^−11^
	Rank	1	3	8	5	9	7	4	2	6
F30	Best	8.10 × 10^3^	8.03 × 10^4^	6.04 × 10^7^	2.36 × 10^7^	7.06 × 10^8^	3.94 × 10^8^	7.08 × 10^6^	2.31 × 10^5^	5.79 × 10^8^
	Std	6.29 × 10^3^	6.74 × 10^4^	1.10 × 10^9^	1.39 × 10^8^	1.65 × 10^9^	2.27 × 10^9^	4.37 × 10^7^	3.11 × 10^5^	2.06 × 10^9^
	Avg	1.40 × 10^4^	1.80 × 10^5^	6.47 × 10^8^	8.55 × 10^7^	2.98 × 10^9^	3.46 × 10^9^	4.94 × 10^7^	6.99 × 10^5^	4.03 × 10^9^
	P		3.02 × 10^−11^	3.02 × 10^−11^	3.02 × 10^−11^	3.02 × 10^−11^	3.02 × 10^−11^	3.02 × 10^−11^	3.02 × 10^−11^	3.02 × 10^−11^
	Rank	1	2	6	5	7	8	4	3	9

**Table A3:** Five types of data test set signals for bearing fault feature frequencies.

Simulated Signal of Inner Ring Fault	Simulated Signal of Outer Ring Fault	CWRU Signal of Inner Ring Fault	CWRU Signal of Outer Ring Fault	Nanchang Railway Bureau Signal of Outer Ring Fault
120 Hz	100 Hz	162 Hz	107 Hz	61 Hz

## References

[1] Tao H., Qiu J., Chen Y., Stojanovic V., Cheng L. (2023). Unsupervised cross-domain rolling bearing fault diagnosis based on time-frequency information fusion. Franklin Inst..

[2] Li Y., Xu M., Wei Y., Huang W. (2015). An improvement EMD method based on the optimized rational Hermite interpolation approach and its application to gear fault diagnosis. Measurement.

[3] Lei Y., He Z., Zi Y. (2009). Application of the EEMD method to rotor fault diagnosis of rotating machinery. Mech. Syst. Signal Process..

[4] Ren Y., Suganthan P.N., Srikanth N. (2014). A comparative study of empirical mode decomposition-based short-term wind speed forecasting methods. IEEE Trans. Sustain. Energy.

[5] Qian P., Ma E., Wang Y., Li Q., Xu H., Zhang D., Tian X. (2025). A novel multi-band demodulation method and its application in bearing fault detection. Measurement.

[6] Wen J., Ren J., Zhao Z., Chen X. (2025). Robust Anomaly Detection of Rotating Machinery with Contaminated Data. J. Dyn. Monit. Diagn..

[7] Sprito M., Nicolella A., Melluso F., Maffi P., Cosenza C., Savino S., Niola V. (2025). Enhancing SDP-CNN for Gear Fault Detection Under Variable Working Conditions via Multi-Order Tracking Filtering. J. Dyn. Monit. Diagn..

[8] Wang Z., Xuan J., Shi T. (2024). Domain reinforcement feature adaptation methodology with correlation alignment for compound fault diagnosis of rolling bearing. Expert Syst. Appl..

[9] Wang Z., Xuan J., Shi T. (2024). An autonomous recognition framework based on reinforced adversarial open set algorithm for compound fault of mechanical equipment. Mech. Syst. Signal Process..

[10] Dragomiretskiy K., Zosso D. (2013). Variational mode decomposition. IEEE Trans. Signal Process..

[11] Liu G., Ma Y., Wang N. (2024). Rolling Bearing Fault Diagnosis Based on SABO–VMD and WMH–KNN. Sensors.

[12] Zhong J., Liu Z., Bi X. (2024). Partial discharge signal denoising algorithm based on aquila optimizer–variational mode decomposition and k-singular value decomposition. Appl. Sci..

[13] Miao Y., Zhang B., Li C., Lin J., Zhang D. (2022). Feature mode decomposition: New decomposition theory for rotating machinery fault diagnosis. IEEE Trans. Ind. Electron..

[14] Wang J., Yuan Y., Shen F., Chen C. (2025). Motor Fault Diagnosis Under Strong Background Noise Based on Parameter-Optimized Feature Mode Decomposition and Spatial–Temporal Features Fusion. Sensors.

[15] Yan X., Jia M. (2022). Bearing fault diagnosis via a parameter-optimized feature mode decomposition. Measurement.

[16] Abdel-Basset M., Mohamed R., Abouhawwash M. (2024). Crested Porcupine Optimizer: A new nature-inspired metaheuristic. Knowl.-Based Syst..

[17] Wang H., Xie J. (2025). Fault Diagnosis of Rolling Bearings Based on Acoustic Signals in Strong Noise Environments. Appl. Sci..

[18] Li M., Yu X., Fu B., Wang X. (2023). A modified whale optimization algorithm with multi-strategy mechanism for global optimization problems. Neural Comput. Appl..

[19] Zhang T., Yin Q., Li S., Guo T., Fan Z. (2025). An Optimized Genetic Algorithm-Based Wavelet Image Fusion Technique for PCB Detection. Appl. Sci..

[20] Feng S., Xia L., Yang Y., Wang Z., Zhang X., Han Q. (2025). Multi-objective collaborative rapid dual-scale topology optimization based on thermomechanical coupling analysis. Arch. Appl. Mech..

[21] Liu J., Wang Y., Fan N., Wei S., Tong W. (2019). A convergence-diversity balanced fitness evaluation mechanism for decomposition-based many-objective optimization algorithm. Integr. Comput.-Aided Eng..

[22] Wu Y., Wang L., Li R., Chen J.F. (2024). A reinforcement learning-driven adaptive decomposition algorithm for multi-objective hybrid seru system scheduling considering worker transfer. Swarm Evol. Comput..

[23] Jia H., Lu C. (2024). Guided learning strategy: A novel update mechanism for metaheuristic algorithms design and improvement. Knowl.-Based Syst..

[24] Ma Z., Yuan X., Han S., Sun D., Ma Y. (2019). Improved chaotic particle swarm optimization algorithm with more symmetric distribution for numerical function optimization. Symmetry.

[25] Guo Y., Yang Y., Jiang S., Jin X., Wei Y. (2022). Rolling bearing fault diagnosis based on successive variational mode decomposition and the EP index. Sensors.

